# Facilitated Monocyte-Macrophage Uptake and Tissue Distribution of Superparmagnetic Iron-Oxide Nanoparticles

**DOI:** 10.1371/journal.pone.0004343

**Published:** 2009-02-02

**Authors:** Arnaud Beduneau, Zhiya Ma, Cassi B. Grotepas, Alexander Kabanov, Barrett E. Rabinow, Nan Gong, R. Lee Mosley, Huanyu Dou, Michael D. Boska, Howard E. Gendelman

**Affiliations:** 1 Department of Pharmacology and Experimental Neuroscience, University of Nebraska Medical Center, Omaha, Nebraska, United States of America; 2 Department of Pharmaceutical Sciences, University of Nebraska Medical Center, Omaha, Nebraska, United States of America; 3 Department of Radiology, University of Nebraska Medical Center, Omaha, Nebraska, United States of America; 4 Center for Neurovirology and Neurodegenerative Disorders, University of Nebraska Medical Center, Omaha, Nebraska, United States of America; 5 Center for Drug Delivery and Nanomedicine, University of Nebraska Medical Center, Omaha, Nebraska, United States of America; 6 Baxter Healthcare Corporation, Round Lake, Illinois, United States of America; Massachusetts General Hospital and Harvard Medical School, United States of America

## Abstract

**Background:**

We posit that the same mononuclear phagocytes (MP) that serve as target cells and vehicles for a host of microbial infections can be used to improve diagnostics and drug delivery. We also theorize that physical and biological processes such as particle shape, size, coating and opsonization that affect MP clearance of debris and microbes can be harnessed to facilitate uptake of nanoparticles (NP) and tissue delivery.

**Methods:**

Monocytes and monocyte-derived macrophages (MDM) were used as vehicles of superparamagnetic iron oxide (SPIO) NP and immunoglobulin (IgG) or albumin coated SPIO for studies of uptake and distribution. IgG coated SPIO was synthesized by covalent linkage and uptake into monocytes and MDM investigated related to size, time, temperature, concentration, and coatings. SPIO and IgG SPIO were infused intravenously into naïve mice. T_2_ measures using magnetic resonance imaging (MRI) were used to monitor tissue distribution in animals.

**Results:**

Oxidation of dextran on the SPIO surface generated reactive aldehyde groups and permitted covalent linkage to amino groups of murine and human IgG and F(ab')_2_ fragments and for Alexa Fluor® 488 hydroxylamine to form a Schiff base. This labile intermediate was immediately reduced with sodium cyanoborohydride in order to stabilize the NP conjugate. Optical density measurements of the oxidized IgG, F(ab')_2_, and/or Alexa Fluor® 488 SPIO demonstrated ∼50% coupling yield. IgG-SPIO was found stable at 4°C for a period of 1 month during which size and polydispersity index varied little from 175 nm and 200 nm, respectively. *In vitro*, NP accumulated readily within monocyte and MDM cytoplasm after IgG-SPIO exposure; whereas, the uptake of native SPIO in monocytes and MDM was 10-fold less. No changes in cell viability were noted for the SPIO-containing monocytes and MDM. Cell morphology was not changed as observed by transmission electron microscopy. Compared to unconjugated SPIO, intravenous injection of IgG-SPIO afforded enhanced and sustained lymphoid tissue distribution over 24 hours as demonstrated by MRI.

**Conclusions:**

Facilitated uptake of coated SPIO in monocytes and MDM was achieved. Uptake was linked to particle size and was time and concentration dependent. The ability of SPIO to be rapidly taken up and distributed into lymphoid tissues also demonstrates feasibility of macrophage-targeted nanoformulations for diagnostic and drug therapy.

## Introduction

The ability of monocytes to migrate to the sites of inflammation, infection, and tissue degeneration makes them attractive vehicles to deliver contrast agents for diagnosis or drugs as therapeutic modalities [1]. Indeed, the tissue distribution of circulating monocytes during inflammatory conditions closely parallels sites of microbial infection [2]. Our laboratory has pursued the question as to whether monocytes and monocyte-derived macrophages (MDM) that disseminate virus in an infected human host could be used to traffic drugs to tissue sites and combat active HIV replication [3], [4], [5], [6]. Precedents for cell-based systems already have been provided for viral gene and drug delivery systems [7], [8], [9]. However, obstacles in realizing this goal center on both cell uptake of drug and monocyte trafficking to tissues known as sites of disease [10]. On balance, progress was, in part, achieved towards these ends in our laboratories. *First*, we demonstrated cell uptake and distribution of nanoparticles (NP) loaded with anti-retroviral medicine [5]. *Second*, we demonstrated that after *ex vivo* loading of monocytes and MDM with NP, the cells readily distribute NP within and throughout body tissues [3]. *Third*, we developed a number of bioimaging, high performance liquid chromatographic, and histological approaches that can readily measure both drug and monocyte-macrophage trafficking and distribution [4], [11]. However, one very important component of this process was lacking; the kinetics of NP uptake and distribution *de novo*. Indeed, if any drug is to be used in the clinic, it need be formulated for parenteral use. Achieving this has proven difficult, as any NP given would be rapidly distributed to the liver, where it would be metabolized and degraded.

The numbers of studies that explore such distributions are quite limited [12]. In the current report, we took advantage of known monocyte functions to facilitate NP uptake. Indeed, it is well known that opsonization of particles and microbes facilitates rapid uptake into monocytes and MDM. We reasoned that NP coating in a similar manner could achieve similar outcomes. As approximately 55% of macrophages in lymphoid tissues are derived from monocyte extravasations [4], we reasoned that if circulating monocytes could take up immunoglobulin (IgG)-conjugated NP after intravenous injection, then particle size and configuration would facilitate monocyte-macrophage uptake. At least one “theoretical” report of mathematical models on the path of macrophages from blood and their potential as vehicles for drug delivery was previously reported [13]. Supporting this idea is a study demonstrating the accumulation of superparamagnetic iron oxide (SPIO)-laden NP in monocytes into sites of neuroinflammation [14]. A number of reasons illustrate the significant advantage for cell-based NP delivery of foreign particles. *First*, monocytes are phagocytic cells and the uptake of micro- and nanosized drug carriers has widely been demonstrated [15], [16]. *Second*, direct intravenous injection of NP can facilitate uptake by circulating monocytes. This approach requires the targeting of monocytes using ligands that interact with antigens or receptors located on the immune cell membrane. The opsonization of foreign particles could facilitate the phagocytic process due to the interaction with Fc receptors highly expressed on the surface of monocytes [17], [18]. *Third*, a cell-based system can achieve carriage of the NP and limit sequestration in organs of the reticuloendothelial system such as the liver. Covalent conjugation of IgG on the SPIO surface affects accelerated uptake of NP in monocytes and enhanced *in vivo* retention without affecting monocyte viability. *Fourth*, attachment of ligands did not induce significant variation of the NP surface charge as was demonstrated with albumin coatings. Our results, taken together, demonstrate the feasibility of coated NP for drug delivery into lymphoid tissues. The ability of magnetic resonance imaging (MRI) to support the feasibility of macrophage-based drug delivery systems renders these tests useful for tracking NP therapeutics in human disease.

## Methods

### SPIO NP and immunoglobulin coatings

Sodium periodate, sodium cyanoborohydride, neocuproine, ammonium acetate, ascorbic acid and potassium permanganate were purchased from Sigma-Aldrich, St. Louis, MO. Murine and human IgG F(ab')_2_ and Fc fragments were purchased from Jackson ImmunoResearch Laboratories, Inc., West Grove, PA. Rat IgG2a anti- MsCD16/CD32 (FcgRIII/FcgRII) (2.4G2), Rat IgG2a anti-trinitrophenol (TNP) isotype control, IgG1 anti-Hu CD16 (FcgRIII) (3G8), IgG1 anti-Hu CD32 (FcgRII) (3D3), IgG1 anti-TNP (107.3) were purchased from BD Biosciences, San Jose, CA. IgG1 anti-Hu CD64 (FcgRI) (10.1) was purchased from eBioscience, San Diego, CA. Alexa Fluor® 488 carboxylic acid, succinimidyl ester and Alexa Fluor® 488 hydroxylamine were purchased from Invitrogen. Ferumoxides (Feridex IV®, Berlex Laboratories, Wayne, NJ) with an average hydrodynamic size of 150 nm and concentration of iron at 11.2 mg/mL were used as SPIOFeridex® (SPIO) (Bayer Healthcare Pharmaceuticals, Wayne, NJ) and composed of 11.2 mg of iron per ml of aqueous matter. NP were concentrated using a Microcon YM-30 centrifugal filter unit (Millipore, Billerica, MA) and dialyzed against acetate buffer (0.1 M, pH 5.5) overnight at 4°C. Dextran T-10 surrounding the iron core of SPIO was oxidized by reaction with 10 mM sodium metaperiodate in the dark for 1 hour at room temperature. To remove excess reagent, oxidized SPIO were dialyzed against phosphate-buffered saline (PBS) overnight at 4°C. Human IgG (Baxter Heathcare Corporation, Westlake Village, CA) or murine IgG (Jackson ImmunoResearch Laboratories, Inc.) were added to the oxidized NP suspension in PBS with 50 mM of sodium cyanoborohydride in 1 M NaOH at final concentrations of IgG and SPIO of 2 mg/ml and 10 mg/ml, respectively. The incubation was performed overnight under gentle stirring at room temperature and the reaction was quenched by addition of 50 mM Tris-HCl. Free IgG was removed from the NP using Sepharose CL-4B column. Particle size was measured by dynamic light scattering. The presence of IgG covalently attached to NP and free IgG amount were estimated using the micro bicinchoninic acid (BCA) assay (Pierce Biotechnology, Rockford, IL). In parallel preparations, Alexa Fluor® 488 hydroxylamine, mouse IgG and mouse and human IgG F(ab')_2_ fragments were reacted with aldehyde SPIO at room temperature in PBS buffer to obtain control reagents for Fc blocking studies and to synthesize combined fluorescence and antibody conjugated SPIO preparations. For fluorescent nanoformulations, Alexa Fluor® 488 carboxylic acid succinimidyl ester was conjugated with IgG SPIO in carbonate-bicarbonate buffer for 2 h. The free dye was separated from conjugates using PD-10 columns.

### Monocyte, MDM, and bone marrow-derived macrophage (BMM) isolation and cultivation

Human monocytes were obtained by leukopheresis from HIV-1 and hepatitis seronegative donors and were purified by counter-current centrifugal elutriation. Wright-stained cytospins were prepared and cell purity assayed by immunolabeling with anti-CD68 (clone KP-1). To generate MDM, elutriated monocytes were cultivated for up to seven days at a concentration of 2×10^6^ cells/ml at 37°C in a humidified atmosphere in Dulbecco's modified Eagles medium (DMEM) supplemented with 10% heat-inactivated pooled human serum, 1% glutamine, 50 µg/ml gentamicin, 10 µg/ml ciprofloxacin and 1000 U/ml recombinant human macrophage colony stimulating factor (MCSF), a generous gift of Wyeth Inc., Cambridge, MA [3]–[5]. Male BALB/c mice (Charles River Laboratory, Inc., MA), 4–5 weeks of age were used as BMM donors. The femur was removed, the bone marrow expelled, cells dissociated into single cell suspensions and cultured for 10 days supplemented with 1000 units/ml of MCSF (Wyeth, Inc.). Cultured BMM proved to be 98% CD11b^+^ by flow cytometric analysis using a FACS Calibur flow cytometer (BD Biosciences).

### Studies of formulated SPIO uptake into monocytes and macrophages

For study, human monocytes or MDM were transferred in 8-well Lab-Tek II chamber slides (0.5×10^6^ cells/well). SPIO were added to a final iron concentration of 0.5 mg/ml and incubated for 1 hr at 37°C. After three washings, cells were fixed with 4% paraformaldehyde for 30 minutes at room temperature. For the Fc blocking studies human IgG Fc fragment or anti-Fc receptor antibodies (clones 3G8, 3D3 and 10.1) (10 ug/ml) were added to MDM cultures, incubated at 37°C for 20 minutes followed by the incubation of SPIO, IgG-SPIO, or F(ab')_2_-SPIO to a final iron concentration of 0.5 mg/ml for 0.5, 1, 2 and 4 hours. Cells with media alone and with anti-TNP isotype control, 107.3 served as controls. For murine BMM, the procedure was identical to the human cells but mouse IgG Fc fragment and IgG FcgRIII/FcgRII were used as Fc blockers. After 2× PBS washes cells were observed by fluorescence microscopy. The iron content was estimated by ferrozine (see below). Presence of iron was observed by Prussian blue staining with 5% potassium ferrocyanide and 5% hydrochloric acid. After three washings, cells were counterstained with nuclear fast red. Viability of monocytes and MDM was estimated by a live/dead assay (Axxora, LLC, San Diego, CA). Iron labeled monocytes were incubated with a mixture of 0.5 µM of ethidium homodimer-1 with 1 µM of calcein AM at 37°C under humidified atmosphere for 25 minutes. The cells were washed three times, fixed with 4% paraformaldehyde for 30 minutes at room temperature, and observed by fluorescent microscopy. Calcein-AM accumulation and cleavage by cytosolic esterase labels live cells green and ethidium homodimer-1 labels the nuclei of dead cells red. Cell viability was confirmed by trypan blue exclusion wherein iron-labeled human monocytes were incubated with trypan blue for 15 minutes and washed three times. Unlabeled cells and human monocytes that were killed using a 70% methanol solution served as controls. For transmission electron microscopy (TEM), human monocytes and MDM were cultivated on poly-d-lysine-coated Thermanox coverslips (Thermo Fisher Scientific, Rochester, NY), incubated with SPIO during 1 hour at 37°C, and washed three times with phosphate buffered saline. They were fixed in 2% glutaraldehyde, 2% paraformaldehyde, 0.5% acrolein in 0.1 M Sorensen's phosphate buffer pH 7.2, and were post-fixed in 1% osmium tetroxide in water for 30 minutes. After washing, human monocytes were dehydrated for 5 minutes at each step in a graded series of ethanol solutions and infiltrated with araldite embedding media by passing through a graded series of ethanol/araldite solutions. Coverslips were embedded culture side face down onto blank araldite discs, placed overnight in an oven at 65°C for polymerization, and removed by dipping in liquid nitrogen. Monocyte and MDM colonies were cut out and mounted *en face* for ultra thin sectioning. Sections were placed on 200 mesh copper grids, and grids were stained with 1% uranyl acetate and Reynold's lead citrate. Cells were examined with a Philips LS410 TEM operated at 80 Kv.

Iron content was estimated using a colorimetric ferrozine assay [19]. For this assay, duplicate batches of human monocytes and MDM were cultivated in 24-well plates (1×10^6^ cells/well). SPIO were incubated at a final concentration of 0.5 mg/ml for 0.5, 1, 2, 4 and 8 hours, at 4°C and 37°C. Iron-labeled cells were washed three times with PBS and lysed with 50 mM NaOH for 1 hr at room temperature on a shaker. Aliquots of cell lysates were mixed at equal volumes with 10 mM hydrochloric acid (HCl) in order to dissolve the SPIO. An iron releasing-reagent, a mixture of 1.4 M HCl and 4.5% (w/v) KMNO_4_ in distilled water, was added to the lysates and the mixtures were incubated for 2 hour at 60°C. Ferrozine assay reagents were prepared in distilled water from 6 mM ferrozine, 2.5 M ammonium acetate, 1 M ascorbic acid and 6.5 mM neocuproine pre-dissolved in methanol. The samples were transferred to a 96-well plate and read at 540 nm. Iron standards were prepared under the same reaction conditions using a stock solution of iron at 0.2 mg/ml (Ricca Chemical Company, Arlington, TX). Iron concentration values were normalized to protein concentrations as determined by the micro BCA assay (Pierce Biotechnology). T_2_ relaxivity measurements of iron-labeled human monocytes were also performed by MRI analysis of triplicate samples for each time-point. SPIO-labeled cell phantoms were prepared as 100 µl of 0.5×10^6^ cells/ml suspended in 100 µl 2% agarose in 200 µl plastic tubes.

### Animal studies for SPIO and IgG SPIO tissue distribution

Male BALB/c mice (Charles River Laboratory, Inc., Wilmington, MA), 5–8 weeks old were used for all experiments. Animals were housed in sterile microisolator cages and maintained in accordance with ethical guidelines for the care of laboratory animals of University of Nebraska Medical Center and the National Institutes of Health. Mice were injected with SPIO or IgG-SPIO at either 12.5 µg/0.2 ml/mouse or 62.5 µg/0.2 ml/mouse intravenously via the tail vein. The recommended dose for a human adult or adolescent is 560 µg/kg. Two groups of mice with 3–5 animals per group were used for *in vivo* analyses. SPIO and IgG-SPIO treated groups were imaged before injection, continuously for 4 hours after injection, and again at 24 hours. Mice were injected within 24 hours following preparation of conjugated SPIO.

### MRI for monocyte-macrophage tissue distribution

SPIO particle accumulation in tissue causes an increase in the magnetic spin-spin relaxivity (R_2_) of tissue water, which is field-dependent. Measures of spin-spin relaxivity using two 7T MRI systems (Bruker 21 cm Biospec/16 cm Pharmascan systems operating Paravision 4.0) demonstrated in SPIO-labeled cell phantoms that relaxivity is related directly to cell density. Measures of relaxivity can track cell uptake and has been used to track the migration of cells to liver, kidney and spleen after injection [11]. High-resolution, multislice multi-echo CPMG phase cycled T_2_ mapping MRI scans of mouse body were acquired using a 25-mm birdcage volume coil covering a region from the neck to the hips with acquisition parameters of echo time (TE) = 10, 20, 30, 40, 50, 60, 70, 80 ms, repetition time (TR) = 4650 ms, number of averages (NA) = 4, field of view = 40×40 mm with a resolution of 256×128 (voxel size = 156×312 µm), reconstructed to 256×256, 50 interleaved contiguous 1 mm thick slices, total acquisition time = 39 minutes. Signal intensity was normalized to an external standard to account for signal drift over time. SPIO accumulation was determined by changes in R_2_ (1/T_2_) within selected regions of interest. After the injection of SPIO or IgG SPIO, T_2_ maps were acquired every 40 minutes for 4 hours, and at 24 hours. Intensities of the regions of interest in spleen, liver and kidney from the even numbered echoes (accurately refocused in CPMG phase cycled echo trains) were fit to an exponential decay with a minimum to match the noise level measured in the images. Data quality was monitored by the T_2_ determination of external standards in each set of images.

### Histological evaluations

Spleens and livers were collected at 4 and 24 hours after SPIO or IgG-SPIO administration. Immediately after MRI, tissues were fixed by perfusion with 4% paraformaldehyde, post fixed for 24 hours, embedded in paraffin and cut into 5 µm thick sections for histological analysis. For Prussian blue staining, slide mounted sections were deparaffinized, rehydrated, and reacted for 30 minutes in 2% potassium ferrocyanide and 3.7% hydrochloric acid to visualize ferric iron particles by Prussian blue. Stained sections were washed and counter stained with nuclear fast red to provide histological cellular distributions. Images were obtained by Optronics digital camera (Buffalo Grove, IL) with MagnaFire 2.0 software (Goleta, CA) and processed by Adobe® Photoshop 7.0 software.

## Results

### SPIO Conjugates

**Figure 1:**
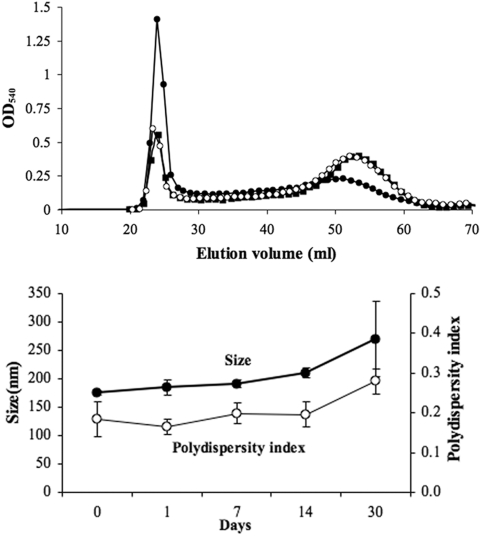
Manufacture, column elution and characterization of IgG SPIO. Top: Sepharose 4B-CL elution profile of IgG-SPIO after incubation of IgG with oxidized SPIO (closed circles). Controls were performed with IgG incubated with non-oxidized SPIO (closed squares) as well as oxidized SPIO blocked by addition of an excess of amine groups (open circles). Bottom: Size and polydispersity index measurements of IgG-SPIO at different time points after the conjugation and storage at 4°C.

The oxidation of dextran located on the surface of SPIO generated reactive aldehyde groups. The carbonyls reacted spontaneously with the amino groups of IgG to form a Schiff base. This labile intermediate was immediately reduced with sodium cyanoborohydride in order to stabilize the bond between the protein and the NP. The IgG-SPIO was then purified using a Sepharose CL-4B column. Alexa Fluor® 488 hydroxylamine was reacted with aldehyde SPIO to synthesize combined fluorescence and IgG conjugated SPIO. The free dye was separated from conjugates using PD-10 columns. BCA reagent was added to each collected fraction and the optical density (OD) was measured at 540 nm. Sepharose CL-4B chromatography of NP conjugate reactions showed that NP eluted between 20 ml and 25 ml and the free IgG between 45 ml and 60 ml (Figure 1, *top*). SPIO reacted with IgG yielded higher BCA reactivity compared to control SPIO due to the presence of increased protein moieties, while the proportion of free IgG in the second peak was lower. The coupling yield was estimated at ∼50%. These differences were not observed when aldehydic groups were blocked with excess amines. Additionally, the elution profile of oxidized and amine-blocked SPIO incubated with IgG was similar to that of free SPIO (data not shown). That the BCA reactivity of eluted SPIO did not approach baseline suggested the possible interference of the dextran coating the SPIO. The stability of IgG-SPIO was studied at 4°C at selected time points for a period of one month by measures of size and polydispersity index (Figure 1, *bottom*). Immediately after conjugation, IgG-SPIO was 175 nm in size and had a polydispersity index of approximately 0.2. No significant changes in hydrodynamic diameter and the size distribution were noticed during the first two weeks. However, 30 days after conjugation, IgG-SPIO increased up to 270 nm and the polydispersity index approximated 0.3 suggesting limited NP aggregation.

### Monocyte uptake of SPIO NP

**Figure 2:**
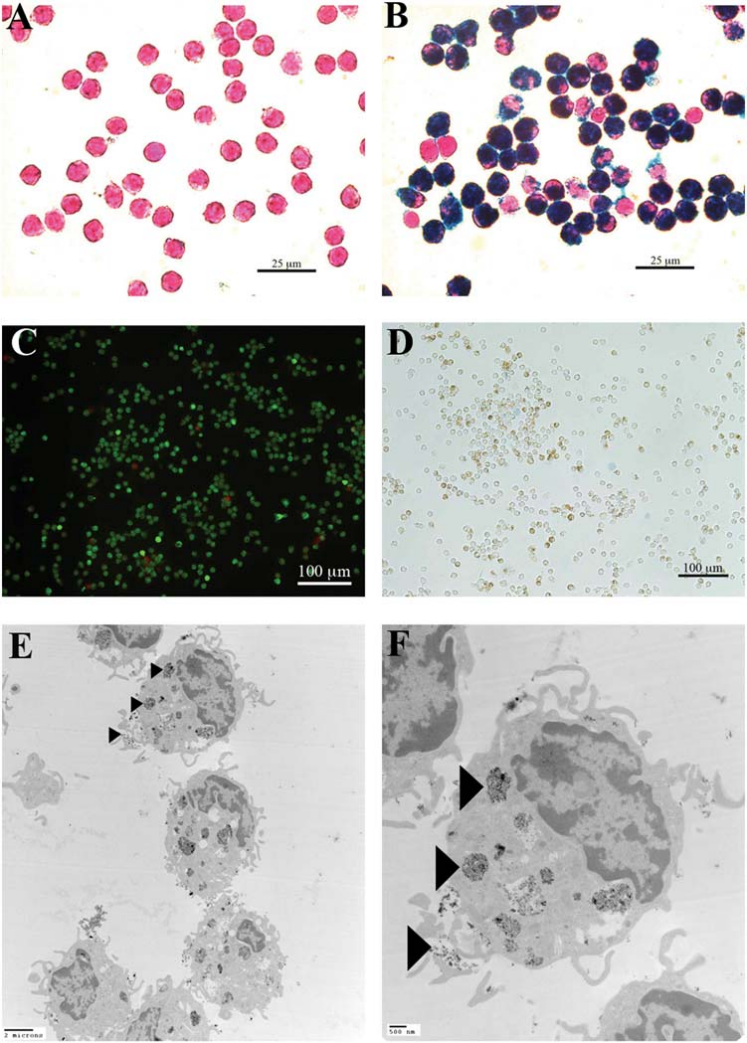
Mechanisms and cell viability for IgG SPIO monocyte uptake. Human monocytes were incubated for 1 hour at 37°C with SPIO (A) or IgG-SPIO (B) at a concentration of 0.5 mg iron/ml. Iron was stained by Prussian blue technique and cells were counterstained with nuclear fast red. Toxicity of IgG-SPIO was evaluated after 1-hour incubation with human monocytes. Viability of iron-labeled monocytes was assessed using a live/dead assay with calcein-AM and ethidium homodimer-1 (EthD1) (C). Calcein-AM accumulation and cleavage by cytosolic esterase labeled live cells in green and EthD1 labeled dead cells in red. Trypan blue exclusion was used as a dual control (D). Internalization of IgG-SPIO was observed by TEM. IgG-SPIO labeled monocytes were observed at 4,400× (E) and 10,400× (F) magnifications. The arrowheads show the SPIO within the cell cytoplasm.

Purified IgG-SPIO and unconjugated SPIO were incubated at 37°C with human monocytes at a final iron concentration of 0.5 mg/ml. Cellular iron content was detected with Prussian blue staining and the cells were counterstained with nuclear fast red (Figure 2A and B). A high accumulation of iron was observed with IgG-SPIO; whereas, the uptake of native SPIO by human monocytes was limited. The viability of iron-containing monocytes was then studied using both a live/dead assay (Figure 2C) and trypan blue staining (Figure 2D). After a 25 minute exposure with calcein-AM and ethidium homodimer-1 (EthD1), human monocyte viability was consistently >90% as characterized by green fluorescence labeling. Similarly most of the iron-labeled monocytes excluded trypan blue confirming that internalization of IgG-SPIO did not induce a toxic effect. Transmission electron microscopy (TEM) was used to study the location of SPIO in human monocytes (Figure 2E and F). Accumulation of chromatin was observed in the nuclear periphery of human monocytes. IgG-SPIO was taken up in cells within endocytic vesicles with size reaching 0.7–1 µm. Distribution of IgG-SPIO was intracytoplasmic with preferential location of NP on small membrane ruffles. Both the number and the size of iron-loaded vesicles were increased in the IgG-SPIO formulations.

**Figure 3:**
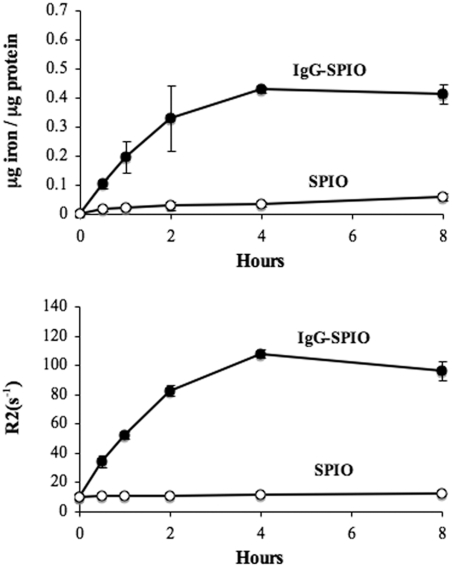
Intracellular iron levels by MRI phantoms after SPIO and IgG-SPIO monocyte uptake. Internalization kinetics of IgG-SPIO and SPIO at iron concentrations of 0.5 mg/ml were determined in human monocytes incubated at 37°C. (A) Estimation of the iron loading by ferrozine assay (top) and by measurement of the relaxation (R2) of iron-labeled monocytes (bottom).

Cellular iron content was assessed using a colorimetric ferrozine assay and by MRI after washing and lysis of cells. Values were normalized by estimation of the protein concentration (Figure 3, *top*). Covalent attachment of human IgG accelerated SPIO uptake as determined by colorimetric assays. After 1 hour, the iron content of cells, ∼0.2 µg iron/µg protein, reached half of the maximum value. In contrast to IgG-SPIO, the internalization of SPIO versus time was linear until 8 hours of incubation, passing from ∼0.03 to 0.06 µg iron/µg protein between 4 and 8 hours. For the majority of times investigated, NP content was nearly an order of magnitude higher than that of cells treated with uncoated NP. In parallel, MRI was used to detect the labeling of monocytes and ensure that the coupling procedure did not alter the NP magnetic properties (Figure 3, *bottom*). Labeled human monocytes were suspended in 2% agarose and the relaxation time T_2_ was measured. In accordance with the previous experiment, the iron uptake was drastically improved when human monocytes were exposed to IgG-SPIO. Maximal levels of SPIO were reached after a 4 hour-incubation. Despite the oxidation step, the relaxation properties of IgG-SPIO were preserved.

**Figure 4:**
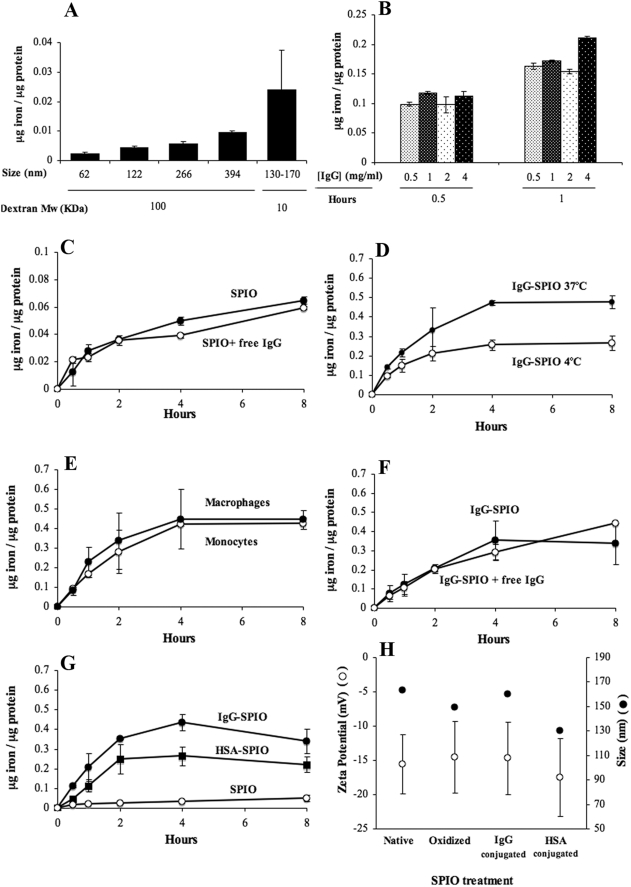
NP size and molecular weight on SPIO uptake into monocytes and MDM. Influence of the size of nanoparticles and the molecular weight of dextran on the uptake of SPIO (A). Uptake by human monocytes of IgG-SPIO with different IgG densities (B) and of native SPIO co-incubated with free IgG (C). Uptake in human monocytes of IgG-SPIO incubated at 4°C and 37°C (D) and comparison between human monocytes and MDM (E). Uptake in human monocytes of IgG-SPIO co-incubated with excess of free IgG (F) and of SPIO conjugated to human serum albumin (HSA) instead of IgG (G). Panel H shows the influence of the oxidation and of the covalent attachment of IgG and HSA on the size and the surface charge of SPIO. Error bars are SEM.

To test the effect of size and dextran coating on cellular uptake, SPIO coated with 100 kDa dextran with sizes ranging from 62 to 394 nm were incubated with human monocytes for 1 hour at 37°C (Figure 4A). A greater iron uptake was detected for the largest NP with the same molecular weight of dextran. However, despite a lower size, SPIO coated with 10 kDa-dextran (Feridex®) were taken up in monocytes at a higher level than other NP coated with 100 kDa-dextran. Considering that the phagocytosis process is mediated by adsorption of serum proteins, the molecular weight of dextran is a crucial parameter for the adsorption of opsonins and consequently, the uptake of NP. In the nanometer range, the size effect on the opsonisation is much less important than the hydrophilic coating. After 1 hour-oxidation, SPIO was conjugated with different concentrations of IgG (0.5, 1, 2 and 4 mg/ml) and each conjugate IgG-SPIO complex was incubated with human monocytes for 0.5 and 1 hr at 37°C. Cellular uptake of IgG-SPIO prepared at different IgG concentrations was not drastically different after incubation with monocytes for 0.5 and 1 hour (Figure 4B). These results suggested that either the cell surface was saturated by SPIO or that internalization is not dependent on ligand density. Non-covalent attachment of IgG did not induce an enhanced uptake for these specific experiments (Figure 4C). Nonetheless, the steric barrier generated by the dextran surrounding the iron core of NP could have prevented adsorption of IgG. IgG-SPIO uptake was partially inhibited when incubation was performed at 4°C (Figure 4D). Next, IgG-SPIO uptake by monocytes and MDM were compared and found to be virtually equal for both cell types (Figure 4E). The results support the idea that similar mechanisms for iron uptake were operative amongst both cell types. In order to study the mechanism of internalization, IgG-SPIO and free IgG, at a concentration of 1 mg/ml, were co-incubated with human monocytes (Figure 4F). No significant differences were detected after the blocking of monocyte Fc receptors with free IgG. These results suggested an Fc receptor-independent mechanism for IgG-SPIO cell entry. To test that possibility, human serum albumin (HSA) was covalently attached to SPIO using the identical oxidation/reduction procedure as IgG conjugation. Similar to IgG, HSA significantly enhanced the uptake of SPIO (Figure 4G). In order to confirm the notion that particle uptake was independent of the Fc receptor we performed blocking studies. Regression analysis of intracellular iron of IgG-SPIO and F(ab')_2_-SPIO uptake by MDM over 4 hours of co-culture showed no differences in uptake kinetics (P = 0.271), indicating that the presence of the IgG Fc portion provided no significant advantage for IgG-SPIO uptake kinetics. Similarly, no differences in IgG-SPIO uptake kinetics were discernable by treatment of MDM with human Fc fragments, anti-Fc receptor-γ antibodies (CD16/CD32/CD64), or isotype control antibody compared to untreated MDM (P = 0.624). Additionally, results in human MDM were validated in mouse BMM wherein no differences were observed in uptake kinetics of mouse IgG-SPIO by BMM treated with mouse Fc fragments, rat anti-mouse Fc receptor-γ (CD16/CD32), or rat isotype control antibody compared to untreated BMM (P = 0.988). These results confirmed the hypothesis that the internalization of IgG-SPIO was not Fc-receptor mediated. Although internalization of HSA-SPIO was similar to IgG-SPIO, the iron content in human monocytes was higher in monocytes incubated with IgG-SPIO than HSA-SPIO. This could be due to the fact that HSA modified the conjugated SPIO itself. To test the latter possibility, physical-chemical properties of SPIO including zeta potential and size measurements of the nanoparticles were performed in HEPES buffer at pH 7.4. HSA modifications of the SPIO did not affect its size (Figure 4H). In addition, the zeta potential of NP did not significantly change after ligand attachment; thus, SPIO remained negatively charged.

### SPIO and IgG SPIO tissue distribution

**Figure 5:**
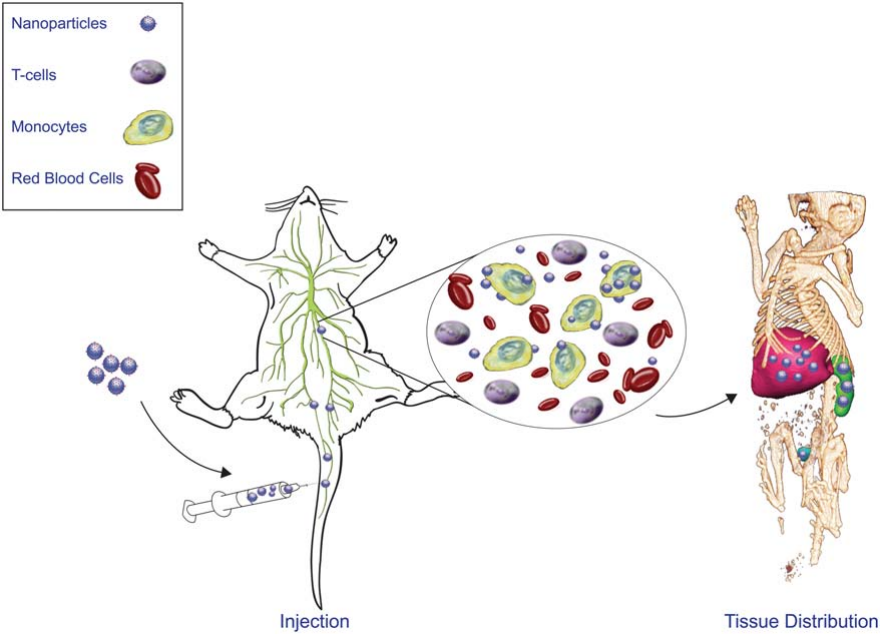
Schematic diagram for IgG SPIO NP cell tissue delivery. We posit that NP can be efficiently taken up by circulating blood borne monocyte-macrophages in ways that are not toxic to the cell function or mobility. The NP loaded cells are then transported to areas of the lymphoid and reticuloendothelial system (for example, liver and spleen) and secreted through the kidneys.

**Figure 6:**
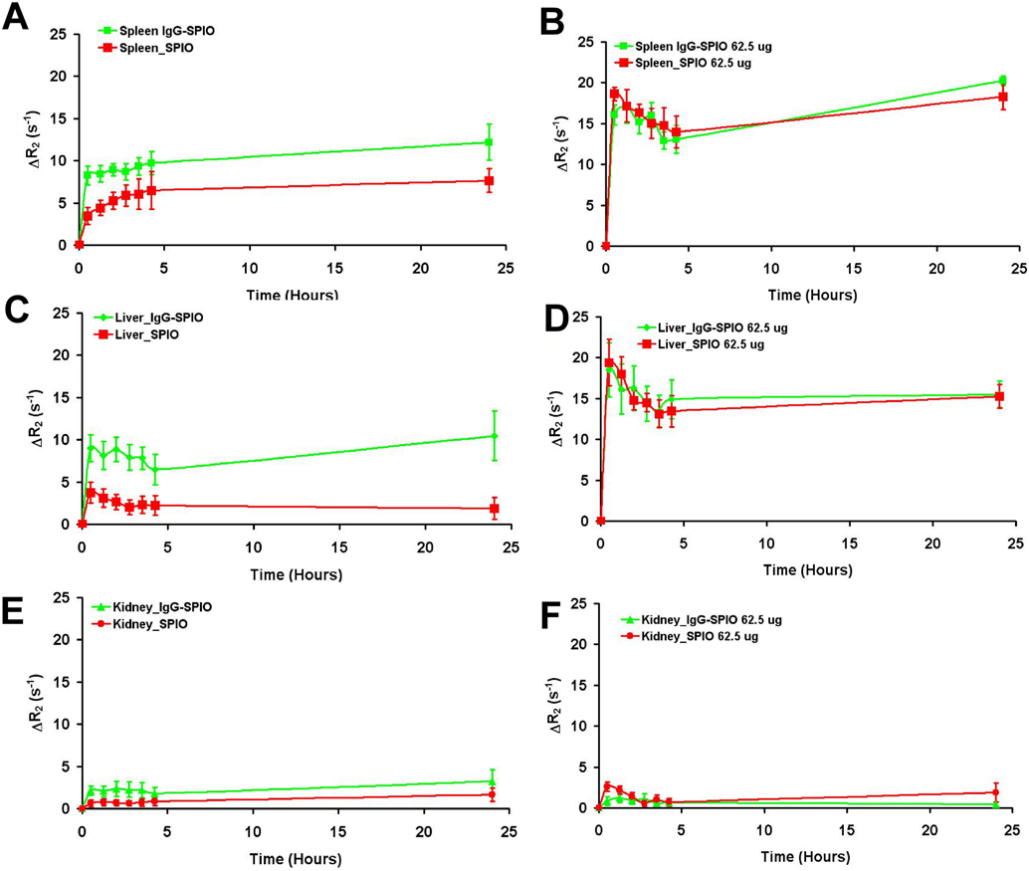
Quantitation of in *vivo* IgG SPIO and SPIO tracking using CPMG phase cycled T_2_ mapping MRI. Change in relaxivity (R_2_ = 1/T_2_) in the spleen (A, B), liver (C, D) and kidney (E, F) after injection of SPIO (red) or IgG-SPIO (green) into the tail vein of the mouse. Injections of 12.5 µg (A, C, E) (n = 6) and 62.5 µg (B, D, F) (n = 5) show higher uptake in circulating monocytes with IgG coating at the lower concentration by 0.5 h post-injection and times thereafter (p<0.05 two way repeated measures ANOVA for effect of time and SPIO type for spleen and liver). At the higher 62.5 µg dosage, no differences in uptake of SPIO vs. IgG-SPIO at any time were observed.

**Figure 7:**
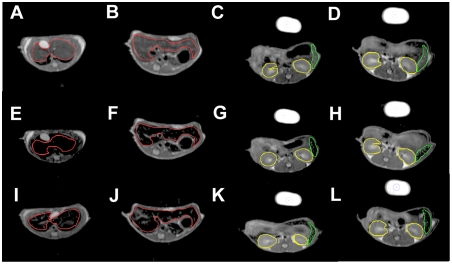
Tracking monocyte-macrophage of IgG SPIO and SPIO migration by MRI. Representative images and regions of interest in single slices showing the signal intensity at TE = 40 ms of MRI scans taken at pre-injection (A–D), 30 minutes after injection (E–H), and 24 hours after injection (I–L). Mice were injected i.v. with 12.5 µg of IgG-SPIO (E,G,I,K) or SPIO injected (F,H,J,L). Regions of interest were selected in the liver (red), (A, B, E, F, I, J), spleen (green) and kidneys (yellow) (C, D, G, H, K, L). Signal loss from the accumulation of SPIO can be seen in the 0.5 hour and 24 hour images.

**Figure 8:**
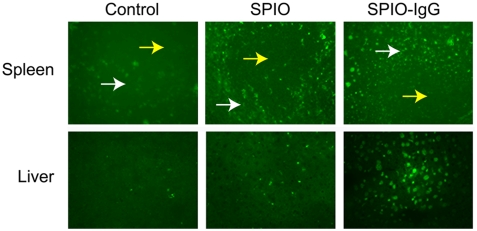
Fluorescently labeled nanoparticles in spleen and liver. Mice were injected i.v. with 12.5 µg of fluorescently labeled SPIO or IgG that was covalently conjugated to SPIO (SPIO-IgG). Twenty four hours after injection animals were sacrificed and spleen and liver were recovered, flash frozen, then cut into 10 µ frozen sections. Tissue sections were fixed with methanol/acetone for 5 minutes and examined by fluorescence microscopy. Spleen and liver from untreated mice served as controls for nonspecific fluorescence. Germinal centers (yellow arrows) and perifollicular areas (white arrows) are denoted within spleen. Magnification, ×400.

To assess comparative tissue distribution of SPIO and IgG-SPIO, we injected NP intravenously into naïve mice. A cartoon of the technique with proposed distributions into lymphoretricular structures is shown in Figure 5. Both IgG-SPIO and Alexa Fluor® 488 hydroxylamine IgG-SPIO nanoformulations were used in study. Following intravenous injection, tissue biodistribution of SPIO was tracked by MRI over the first 4 hours and at 24 hours post-injection. MRI tests revealed both hepatic and splenic accumulations of IgG-SPIO demonstrating higher levels for each post-injection time point in the spleen and liver at lower concentrations of injected NP (12.5 µg, n = 6, Figure 6 A, C, and E) as well as in representative images in Figure 7. However, at the higher concentration (62.5 µg, n = 5), results were identical between SPIO and IgG-SPIO injected mice (Figure 6 B, D, and F), supporting the notion that NP saturated the lymphoreticular system. SPIO were seen distributed in macrophage perifollicular areas in spleen and diffusely in the liver. Uptake of SPIO was seen in circulating blood monocytes (data not shown) and tissue macrophages by analysis of tissue specimens from spleen and liver of animals that were injected with Alexa Fluor® 488 hydroxylamine IgG-SPIO nanoformulations following the MRI tests (Figure 8).

## Discussion

The objective of our research efforts is to substantively improve drug delivery through the use of cell based carriers for tumors and microbial infection, in particular HIV [1]. Our focus has over the past quarter century addressed MP as reservoirs and vehicles for dissemination of virus and as such led us to explore whether the same cells can be used as drug carriers for antiretroviral therapies [22], [23]. In order to test these hypotheses, four critical milestones had to be achieved. *First*, we needed to manufacture NP that could rapidly enter MP and be taken to infected tissue reservoirs. *Second*, the drugs needed to be released from carrier cells in a continuous manner over time periods measured in weeks. *Third*, the uptake, transport, and release needed to occur without notable system toxicity. *Fourth*, sustained transport of cells needed to take place beyond the liver and particle clearance seen in lymphoid tissues including the spleen. To achieve the latter goal, we used a simple means, antibody opsonization, to facilitate uptake of particles into MP; the process by which the host facilitates foreign particles for ingestion and destruction. We reasoned that this process might also be used to coat NP. Indeed, the binding of a pathogen by antibody to a receptor on the cell membrane can promote macrophage uptake through Fc receptors [24]. Alternatively, the complex would stimulate the phagocyte nonspecifically and in this manner membrane alterations would occur without changes in membrane properties to facilitate uptake [25]. The receptor-opsin NP complex [26] would create byproducts that could include C3b and C4b and other components of the complement system. These would be deposited on the cell surface of the antibody linked NP and positively affect its uptake.

Nonetheless and regardless of the mechanism, our results demonstrated that coating of NP enhanced uptake and positively affected NP biodistribution, making the coated NP an attractive candidate for drug delivery. It is likely that circulating monocytes were able to take up NP and as such, deliver them to the spleen rather than simply clearing the particles through the liver. There is precedent for this as monocytes are known to incorporate micro- and nano-sized drug carriers (such as liposomes or nanoparticles) and act as Trojan horses by crossing the brain and enabling drug delivery [27], [28], [29]. Moreover, it was shown that during neurological diseases (such as Parkinson's and Alzheimer's diseases, meningitis, viral encephalitis, Prion disease, and HIV-1 associated neurocognitive disorders) monocyte brain migration can occur based on the generation of chemotactic gradient facilitated by inflammation [30]. This occurs in a broad range of infectious, neoplastic, and degenerative disorders whereby inflammation occurs in tandem with extensive leukocyte recruitment. Monocytes and macrophages have the unique ability to migrate to sites of inflammation through diapedisis and chemotaxis [31]. Other abilities of these cells include phagocytosis of foreign particles, production of cytotoxic compounds, and subsequent release of those compounds by exocytosis making myeloid lineage cells attractive candidates for cell based NP carriage of drug [1], [2].

There are many examples that MP may be used for drug delivery. They are first and foremost capable of endocytosing colloidal nanomaterials and subsequently releasing them into surrounding media [27], [29]. A method used to increase the migration of these cells to the brain is to package magnetic particles along with drug inside liposomes, then apply a local magnetic field to the head [29]. MP seem to prefer negatively charged compounds as they preferentially uptake liposomes containing negatively charged lipids or liposomes modified by poly-anions [28]. This offers a possible strategy to modulate the uptake and release of nanomaterials. Liposomes containing phosphatidylserine (a negatively charged lipid) and loaded with chloroquine were shown to accumulate within macrophage phagolysosomes and greatly increase the therapeutic activity against chronic fungal infection when compared to high doses of free drug [32].

One attribute of immune cells is the ability to home to diseased sites, thus representing the *raison d'etre* to design NP for direct uptake. Immediately after *ex vivo* manipulation and re-implantation, the majority of macrophages accumulate in the lungs, liver, and spleen rather than to the targeted diseased tissue. However, having circulating monocytes uptake NP, migrating successfully to target tissues [33] would allow drug-laden cells to reach the target site. Once the cell loaded with NP has reached the site of interest, drug could be released to the extracellular space or internalized by the target cell. By this method, drugs with intracellular action that are incapable of crossing cell membranes can be assisted in reaching their target cells or tissue. Cellular uptake mechanisms vary according to cell type, physicochemical properties of the compound, and the mechanism of drug activation [34], [35]. Intracellular targeting is feasible through the use of ligands that trigger receptor-mediated endocytosis.

The option we used is to modify the surface of nanocarriers (for example by coating the surface of the particle in IgG) to induce or enhance cell uptake. It has been shown that NP uptake is both concentration and time dependent. For example, PLGA NP were transported into primary endosomes and sorted to either recycling endosomes or secondary endosomes [36]. In the acidic environment of the secondary endosome, the NP surface changes from anionic to cationic leading to the escape of the NP into the cytoplasm. Previous studies showed that when nanoparticles were delivered locally, drug levels in tissues could be sustained for up to 7–14 days [37], [38]. In a study where pentamidine-laden NP were administered to *Leishmania* infected mice, ultrastructural examination showed NP trafficking inside *Leishmania* infected Kupffer cells. In these cells, the NP were located within vacuoles and primary lysosomes, which form into secondary lysosomes. Secondary lysosomes containing NP and parasitophorous vacuoles were observed [39].

Prussian blue staining of tissue sections was performed in liver and spleens of MRI tested animals, but showed discordant results. One potential confounding factor is the elimination of vascular and perivascular cells during perfusion. Indeed, the circulating monocytes and macrophages were the sole contribution to the signal changes observed in the kidney, which did not sequester cells in steady state. It is also possible that oxidation of SPIO during perfusion and tissue processing could prevent binding of Prussian blue. In addition, tissue sampling, especially in the liver where the distribution was noticeably heterogeneous, could result in broad variability in cell numbers assayed from histological sections. As a result, the quantification of Prussian blue in tissue is under further investigation.

Previous studies performed in our laboratory using indinavir (IDV) nanosuspensions highlighted the importance of cell-based drug delivery for HIV treatment [3], [4], [5]. These experiments revealed that anti-retroviral NP laden BMM can carry and release drug in tissue. After intravenous injection of NP-IDV BMM, tissue and sera IDV levels were sustained above the effective dose 50 (ED_50_) and elicited reductions in numbers of virus-infected cells. However, no studies to date have assessed the rapid and sustained success towards the ability of directly administered NP to be taken up by monocytes and macrophages and be distributed to lymphoid sites.

We used SPIO as a standard test nano-probe to study cell based uptake and carriage. The rationale is based on the fact that similarly sized and configured particles could be used for cell based drug delivery to treat a host of degenerative and infectious disorders. SPIO are taken up readily into monocytes and MDM and do distribute to tissue. Indeed, a study reported by Engberink *et al.* [14] showed the accumulation of SPIO-laden monocytes in neuroinflammation sites. Thus, the use of the immune system to target inflamed tissue represents a promising cell-based strategy to transport NP to targeted cells. The other advantage is to prevent interaction of foreign particles with the host environment, thus prolonging their systemic half-life. For optimal utilization of macrophage delivery systems, uptake of NP within minutes must be achieved along with the accurate assessment as to whether cells reach a targeted distribution. In this study, we explored the feasibility of utilizing direct injections. A necessary step toward implementing this strategy required coatings of the NP and elucidation of the charge, size and geometry of the particle itself. These were accomplished in this investigation including the measurement of macrophage migration and homing to ensure the adequacy of reticuloendothelial distribution. In addition, monocytes are phagocytic cells and uptake of micro- and nano-sized drug carriers has widely been demonstrated [29]. We posit that the loading of monocytes could be performed by direct intravenous injection of NP. This approach requires the targeting of monocytes using ligands interacting with antigens or receptors located on the immune cell membrane. Opsonization of foreign particles facilitates the phagocytosis process. Thus, non-specific human IgG was attached on the surface of SPIO. However, IgG-coated nanoparticles also will be recognized by tissue macrophages and rapidly removed from the bloodstream. The carrier should be able to target circulating monocytes, before sequestration in organs of the reticuloendothelial system such as the liver and the spleen. Due to the large number of common antigens and receptors on monocytes and macrophages, this would have been a common confounding issue regardless of ligand utilized. This limitation could limit the clinical utility of nanoparticles for human use and remains an area of active research in our laboratories.

In the current study, IgG was covalently conjugated to SPIO using an oxidation/reduction procedure. A simple incubation of antibodies with SPIO did not allow the adsorption of a significant amount of IgG. This is due to the steric barrier of dextran surrounding the iron core of the SPIO. This protein-resistant effect was dependent on the molecular weight of dextran. Monocyte uptake of SPIO coated with 100 kDa dextran was lower than that of 10 kDa dextran-coated SPIO, even for the smaller sized SPIO. Covalent conjugation of IgG on the SPIO surface accelerated and increased uptake in monocytes by a factor of 10. After a 30 minute culture, iron accumulation of IgG-SPIO was significantly higher than that of native SPIO even after 8 hour co-culture. T_2_ relaxivity measurements of labeled monocytes confirmed the enhanced cellular uptake using IgG coating. Besides, oxidation of dextran did not significantly affect the magnetic properties of SPIO. The rapid uptake suggests that coated nanoparticles could be taken up by monocytes within a few minutes after their intravenous administration. Although internalization of IgG-SPIO by immune cells was diminished at 4°C demonstrating energy dependency, monocyte uptake was not inhibited after blocking Fc receptors with free IgG, thus demonstrating an Fc receptor-independent mechanism. In addition, replacement of IgG by HSA significantly increased NP uptake compared to unconjugated SPIO. The attachment of ligands did not induce a significant variation of the surface charge of NP and consequently cannot improve interaction with scavenger receptors responsible for macrophage uptake of charged nanoparticles. Since free IgG or HSA-SPIO were unable to totally block uptake, the possibility remains for yet undefined mechanisms to account for NP uptake; possibly, one whereby SPIO mediate immune response activation.

Taken together, this report is a first step toward demonstrating feasibility of coated NP for monocyte uptake and drug delivery. Moreover, these results suggest the plausibility of drug-ladened NP uptake by circulating mononuclear cells for distribution of nanoformulated drug to lymphoid tissues. If achieved, such modalities could provide substantive progress toward the potential to improve pharmacokinetics by delivering drugs to tissue sites where disease is operative and as such, reduce morbidity and improve clinical outcomes in affected people. In addition, uptake by circulating mononuclear cells of coated NP tagged with contrast agents will provide a non-invasive method of detecting regions of inflammation using other imaging methods, including single photon emission computed tomography.
